# Ten years risk assessment of atherosclerotic cardiovascular disease using Astro-CHARM and pooled cohort equation in a south Asian sub-population

**DOI:** 10.1186/s12889-020-08472-4

**Published:** 2020-03-27

**Authors:** Tariq Ashraf, Muhammad Naeem Mengal, Atif Sher Muhammad, Asal Khan Tareen, Muhammad Nauman Khan, Khawar Abbas Kazmi, Asif Nadeem, Shakil Sarwar, Zara Bashir, Nadeem Qamar, Musa Karim

**Affiliations:** 1 National Institute of Cardiovascular Diseases (NICVD), Karachi, Pakistan; 2 Combined Military Hospital (CMH) Malir, Karachi, Pakistan; 3 Aga Khan University Hospital, Karachi, Pakistan

**Keywords:** Atherosclerotic cardiovascular disease, Astro-CHARM, Pooled cohort equation, South Asia

## Abstract

**Background:**

Atherosclerotic cardiovascular diseases (ASCVD) are on the rise in low and middle-income countries attributed to modern sedentary lifestyle and dietary habits. This has led to the need of assessment of the burden of at-risk population so that prevention measures can be developed. The objective of this study was to assess ten years risk assessment of ASCVD using Astro-CHARM and Pooled Cohort Equation (PCE) in a South Asian sub-population.

**Methods:**

A total of 386 residents of all six districts of Karachi with no ASCVD were enrolled in the study through an exponential non-discriminative referral snowball sampling technique. The inclusion criteria consisted of age 40 years or above and either gender. Study participants were enrolled after obtaining informed written consent and those study participants who were found to have either congenital heart disease or valvular heart diseases or ischemic heart disease were excluded from the study based on initial screening. For the calculation of 10 years risk of ACVD based on Astro-CHARM and PCE, the variables were obtained including medical history and coronary artery calcium and C-reactive protein measurements.

**Results:**

Mean estimated 10-year risk of fatal or non-fatal myocardial infarction or stroke as per the Astro-CHARM was 13.98 ± 8.01%, while mean estimated 10-year risk of fatal or non-fatal myocardial infarction or stroke as per the PCE was 22.26 ± 14.01%. Based on Astro-CHARM, 11.14% of the study participants were labeled as having high risk, while PCE estimated 20.73% of study participants as having high risk of ASCVD.

**Conclusion:**

Despite the fact that our findings showed substantial differences in ten-year risk of ASCVD between Astro-CHARM and PCE, both calculators can be used to develop a new population and specific risk estimators for this South Asian sub-population. Our study provides the first step towards developing a risk assessment guided decision-making protocol for primary prevention of ASCVD in this population.

## Background

With the ever-increasing global burden of atherosclerotic cardiovascular diseases (ACVD) the need to timely assess the risk of cardiovascular events cannot be more emphasized [1–5]. Once among the most common causes of death in high-income countries only, ACVD has become the leading cause of morbidity and mortality in low and middle-income countries as well owing to epidemiologic transition and changing population dynamics. This trend is more prominent in the South Asian population where (ACVD) is the leading cause of death [6, 7].

A number of risk estimators have been developed to assess the risk of any cardiovascular event in at-risk population. Astro-CHARM and Pooled Cohort Equation (PCE) has been much widely used worldwide however these estimators have been developed in the west and not much evidence is present that how accurately they estimate ACVD in South Asian population [8, 9]. Tariq et al. [10] reported that the Pakistani population showed to have a higher risk of ASCVD compared to whites, blacks, and Hispanic and even South Asians living in USA. Another study conducted in a similar population showed that the burden of premature myocardial infarction to be as high as 12% [11]. In addition, another large multi-centric study from Pakistan attempted to predict the risk of atherosclerotic cardiovascular disease in Pakistani population, however, included subject with already suffered from ASCVD [12]. Such methodological flaws can have serious implications while projecting risk assessment on the general population. There is a scarcity of literature providing estimates of ASCVD risk in resident South Asian population as well as comparison of different risk estimators in this population.

Majority of these studies have used PCE as a risk estimator which has its own limitation that includes overestimating the risk in other nationalities/ ethnicities and in population with less social deformation which requires attention while making medical decisions. To overcome this Astro-CHARM calculation was used (Astronaut Cardiovascular Health and Risk Modification). Data in white, Hispanic & black have been elucidated except for the South Asians [8].

Cardiovascular risk can be estimated as either absolute, relative, lifetime, or recurrent. An internationally agreed-upon guidelines do not exist in this regard [13]. The 10 years risk of coronary events (high, intermediate, or low) can be determined by various risk scores, such as laboratory and non-laboratory Framingham risk score for CVD, systematic coronary risk evaluation, Reynolds risk score, the Lancet chronic diseases risk charts, World Health Organization risk charts, etc., but poor concordance has been reported between the risk score, jeopardizing their clinical utility [14]. ACC/AHA preventive guidelines advocate the use of PCE for the assessment of cardiovascular risk [15] and Astro-CHARM is considered to be a significant improvement over the PCE with the addition of discriminating power of coronary artery calcium (CAC) and hs-CRP [8]. Keeping in mind the ease of adoption of PCE in a resource-limited setting and predictive superiority of Astro-CHARM, we assessed the risk of ASCVD in our population using both modalities.

This study, to the best of our knowledge, attempted to estimate the ten-year risk of ASCVD in general population without any history of ASCVD using both Astro-CHARM and PCE, the aim of this study was to estimate risk and compared both the modalities.

## Methods

This analytical cross-sectional study was conducted among 386 residents of all six districts of Karachi who had no prior history of atherosclerotic cardiovascular disease including stroke. Study was approved by the ethical review committee of the National Institute of Cardiovascular Diseases (NICVD) Karachi, Pakistan (ERC-29/2018). Karachi is the largest metropolitan city of the country with a diverse ethnic distribution, inhabiting around 15 million people with a fair share of all the ethnicities residing in the country. Due to non-availability of the sampling frame for any probability sampling technique, non-probability snowball sampling technique was adopted. Starting with the recruitment of consenting individuals, for all six districts of the city, meeting the inclusion criteria of the study from healthy attendants visiting our center. The exponential non-discriminative referral technique was adopter where every recruited participant was allowed multiple referrals from his/her acquaintances. All the referrals were contacted and directed to a designated desk at the doctor’s office for initial screening and assessment. After a thorough history, physical, and electrocardiographic assessment to rule out underlying atherosclerotic cardiovascular disease, participants of both gender and age between 40 to 65 years were included in this study. Study participants were enrolled after obtaining informed written consent and those study participants who were found to have either congenital heart disease or valvular heart diseases or ischemic heart disease were excluded from the study based on initial screening. Individuals who refused or shown reluctance to accept computed tomography angiography (CTA) or other laboratory assessments such as lipid profile or C-reactive protein (hs-CRP) were excluded after an initial assessment.

Fasting (12 h) lipid profile and hs-CRP level were obtained for all the enrolled patients and for the calculation of 10 years risk of ACVD based on Astro-CHARM and PCE, the variables were obtained in the following manner. Diabetes mellitus, hypertension, current smoking, and family history of heart attack were defined as per Astro-CHARM definitions [8].

High-sensitivity C-reactive protein (hs-CRP) levels were measured by using the following assays. MESA, BNII nephelometer (N High Sensitivity CRP; Dade Behring Inc); DHS, Roche/Hitachi 912 System, Tina-quant assay (Roche Diagnostics), alatex-enhanced immunoturbidimetric method; PACC, particleenhancedimmune turbidimetric latex agglutination assay; FHS, enhanced immune turbidimetric high-sensitivity assay (Roche Diagnostics). Computed tomography angiography (CTA) was performed in all the patients and coronary artery calcium (CAC) score was calculated for all the patients.

Data were entered using MS Excel for Windows and analyses were done using STATA 11.0 while 10 years risk of ACVD was calculated using Astro-CHARM and PCE using the online calculators available at www.AstroCHARM.org and www.clincalc.com respectively. Participants were stratified as low-risk and high-risk individuals based on cut-off have of ≥7.5% for both Astro-CHARM and PCE.

Descriptive analyses were conducted by calculating the mean and standard deviation for all continuous variables while frequency and percentages were calculated for all categorical variables. Comparison between male and female participants on continuous variables was made by performing independent sample t-test and categorical variables were compared by applying Chi-square test and *p*-value ≤0.05 as taken as criteria for statistical significance. Risk of ACVD was reported using both equations for the combined study population and was also stratified for different sub-groups.

## Results

Mean age of the study participants was 49 ± 7.10 years with 54% Males. Mean BMI was 28.11 ± 5.38 while 45.6, 15.8, and 14.2% were hypertensive, diabetics and current smokers respectively. Six (2 %) of the study participants had all three co-morbid conditions. Thirty percent had positive family history of MI. Mean CAC was 7.50 ± 14.05 Agatston units. Table 1 shows the baseline socio-demographic and medical characteristics of study participants stratified by gender.

**Table 1:** Baseline socio-demographic and medical characteristics of study participants

Baseline characteristics	Overall ***N*** = 386	Gender		***P***-value
		Male ***N*** = 211	Female ***N***= 175	
**Age** (years)^a^	49 ± 7.11	49.47 ± 7.24	48.43 ± 6.93	0.153
**Body mass index** (kg/m^2^)^a^	28.12 ± 5.39	26.93 ± 4.71	29.55 ± 5.8	< 0.001
**Coronary artery calcium** (Agatston units)^a^	31.57 ± 195.63	44.61 ± 253.95	15.86 ± 79.94	0.121
**Total cholesterol** (mg/dL)^a^	177.65 ± 38.97	174.91 ± 37.02	180.94 ± 41.07	0.131
**HDL cholesterol** (mg/dL)^a^	51.73 ± 36.78	50.93 ± 48.39	52.7 ± 12.85	0.639
**Systolic blood pressure** (mmHg)^a^	138.83 ± 21.23	135.73 ± 20.81	142.57 ± 21.18	0.002
**Hypertensive**				
Yes	176 (45.6%)	78 (36.97%)	98 (56%)	< 0.001
No	210 (54.4%)	133 (63.03%)	77 (44%)	
**Diabetic**				
Yes	61 (15.8%)	34 (16.11%)	27 (15.43%)	0.854
No	325 (84.2%)	177 (83.89%)	148 (84.57%)	
**Current Smoker**				
Yes	55 (14.25%)	50 (23.7%)	5 (2.86%)	< 0.001
No	331 (85.75%)	161 (76.3%)	170 (97.14%)	
**Family History of Myocardial Infarction**				
Yes	118 (30.57%)	66 (31.28%)	52 (29.71%)	0.74
No	268 (69.43%)	145 (68.72%)	123 (70.29%)	

^a^ reported as mean and standard deviation, all other variables are reported as frequencies and percentages

**Fig. 1:**
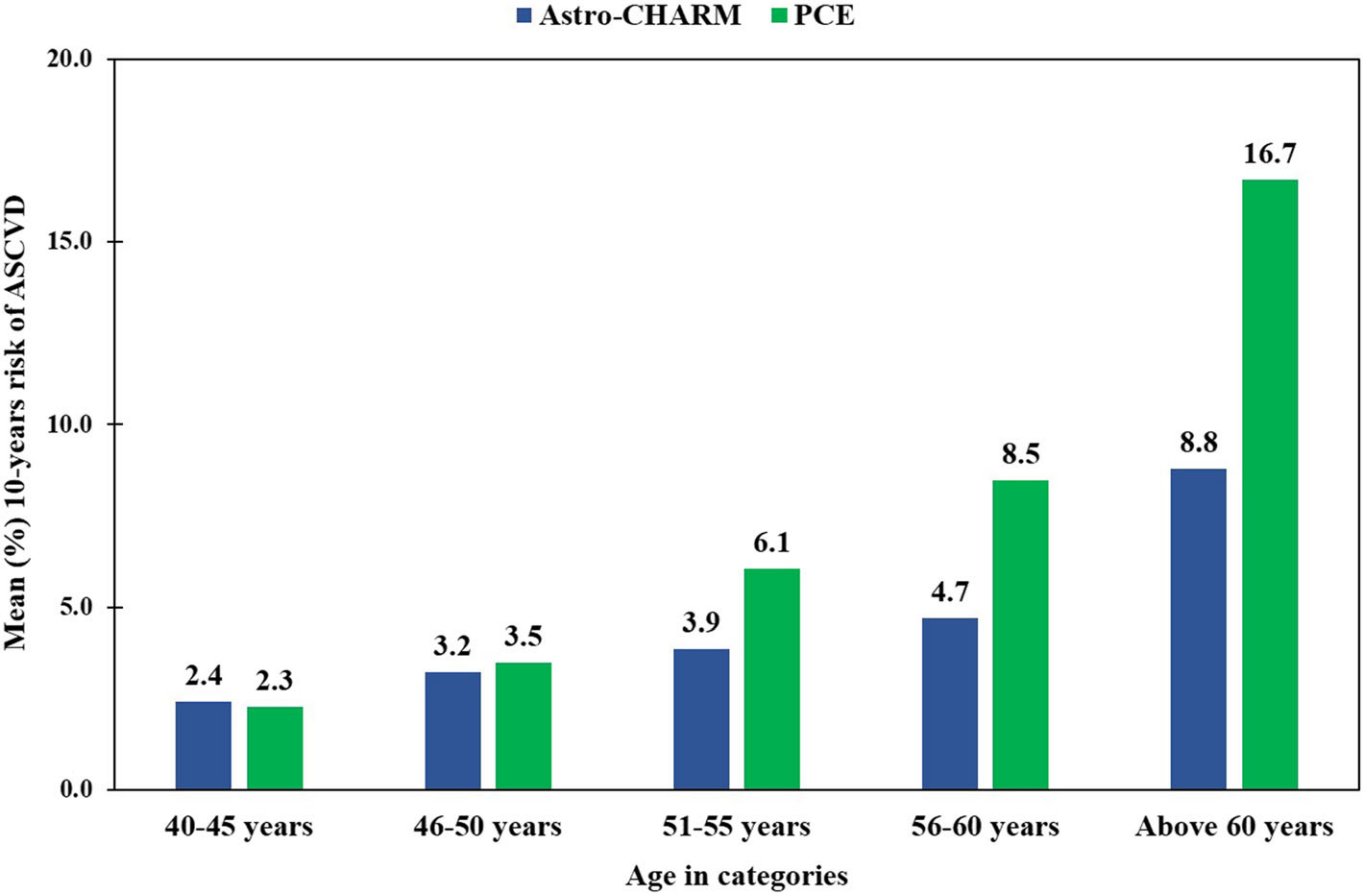
The comparison of mean 10-years risk of ASCVD for different age groups as calcualted by Astro-CHARM and PCE

Mean estimated 10-year risk of fatal or non-fatal myocardial infarction or stroke as per the Astro-CHARM was 13.98 ± 8.01%, while mean estimated 10-year risk of fatal or non-fatal myocardial infarction or stroke as per the PCE was 22.26 ± 14.01%. Risk comparison in different age categories showed a significantly higher risk of ACVD in age groups of 55–60 years and 60 years and above compared to the ASCVD risk in the age group 40–45 years as shown in Fig. 1.

Similarly, Table 2 below shows the comparison of baseline characteristics of high and low-risk individuals based on Astro-CHARM and Pooled Cohort Estimator. Kappa agreement between the two calculators in terms of labeling high and low-risk individuals was high (0.50, *p*-value < 0.0001).

**Table 2:** Comparison of baseline characteristics of high and low risk individuals based on Astro-CHARM and Pooled Cohort Estimator

Characteristics	Astro-CHARM		PCE	
	***Low Risk*** *N = 343*	***High Risk*** *N = 43*	***Low Risk*** *N = 306*	***High Risk*** *N = 80*
**Gender**				
Male	51.9% (178)	76.7% (33)	47.4% (145)	82.5% (66)
Female	48.1% (165)	23.3% (10)	52.6% (161)	17.5% (14)
**Weight** (kg)	73 ± 15	74 ± 14	74 ± 15	72 ± 13
**Height** (cm)	162 ± 11	165 ± 10	161 ± 11	166 ± 10
**Age** (years)	48 ± 7	55 ± 7	47 ± 6	57 ± 6
**Hypertensive**				
Yes	44.6% (153)	53.5% (23)	44.4% (136)	50% (40)
No	55.4% (190)	46.5% (20)	55.6% (170)	50% (40)
**Diabetic**				
Yes	11.1% (38)	53.5% (23)	8.8% (27)	42.5% (34)
No	88.9% (305)	46.5% (20)	91.2% (279)	57.5% (46)
**Current Smoker**				
Yes	11.1% (38)	39.5% (17)	9.2% (28)	33.8% (27)
No	88.9% (305)	60.5% (26)	90.8% (278)	66.2% (53)
**Family History of Myocardial infarction**				
Yes	28.6% (98)	46.5% (20)	32% (98)	25% (20)
No	71.4% (245)	53.5% (23)	68% (208)	75% (60)
**Coronary Artery Calcium** (Agatston)	9.9 ± 46.42	204.2 ± 546.76	15.5 ± 73.87	93 ± 400.79
**Total Cholesterol** (mg/dL)	178 ± 37	178 ± 50	175 ± 39	187 ± 37
**HDL Cholesterol** (mg/dL)	53 ± 39	45 ± 10	53 ± 41	45 ± 11
**Systolic Blood Pressure** (mmHg)	137 ± 20	155 ± 21	137 ± 20	148 ± 22

*Astro-CHARM* Astronaut Cardiovascular Health and Risk Modification*; PCE Pooled Cohort Equation*

*Low-Risk = < 7.5% estimated 10 years risk; High-Risk = ≥7.5% estimated 10 years risk*

Out of 386 individuals, 8.8% (34) were categorized as high risk by both Astro-CHARM and PCE and 76.9% (297) were categorized as low risk by both Astro-CHARM and PCE. Taking Astro-CHARM as gold standard for categorizing high risk individuals, PCE has sensitivity of 79.07%, specificity of 86.59%, positive predictive value of 42.50%, and negative predictive value of 97.06%, as presented in Table 3.

**Table 3:** Cross tabulation of Pooled Cohort Estimator against Astro-CHARM for categorizing high risk individuals

	Astro-CHARM	
	*Low Risk*	*High Risk*
**PCE**		
*Low Risk*	76.9% (297)	2.3% (9)
*High Risk*	11.9% (46)	8.8% (34)
Sensitivity	79.07% (95% CI: 63.96 to 89.96%)	
Specificity	86.59% (95% CI: 82.52 to 90.01%)	
Positive predictive value	42.50% (95% CI: 35.16 to 50.19%)	
Negative predictive value	97.06% (95% CI: 94.85 to 98.34%)	

*Astro-CHARM* Astronaut Cardiovascular Health and Risk Modification*, PCE* Pooled Cohort Equation*, CI* Confidence interval

*Low-Risk = < 7.5% estimated 10 years risk; High-Risk = ≥7.5% estimated 10 years risk*

## Discussion

To the best of our knowledge, current study reports 10 year risk of ASCVD among non-cardiac Pakistani population using both Astro-CHARM and PCE and the comparison of the two calculators for the first time. In concordance with the findings of other studies, PCE estimated the risk of ASCVD in the study population almost twice as that estimated by Astro-CHARM. Astro-CHARM which also includes indicators like coronary artery calcium and C-reactive protein during ASCVD risk assessment, these indicators are not readily available in low and middle-income countries, therefore, the significance of PCE cannot be neglected [16].

One of the key findings of this study was that both coronary artery calcium and C reactive protein significantly correlated with the risk scores generated by PCE which is in concordance with findings from other studies [17, 18]. Especially in the case of CAC, a relatively recent study by JJ Carr et al. [17] reported its effect on the risk of ASCVD among individuals well below 40 years of age. Keeping in mind that said study was conducted among African-Americans and Caucasians, who are relatively at lower risk of developing ASCVD compare to South Asians, it points towards a major limitation of these risk calculators among South Asians, who, in clinical experience suffer cardiac events at an even younger age [19] and evidenced by the previous study from Pakistan [10], there is a dire need of development of population-specific risk estimator for the South Asian population. This study provided the grounds towards developing such risk estimator as well as initial step towards developing evidence-based decision making for primary prevention guidelines of atherosclerotic cardiovascular disease in this population.

Considering the fact that the population of South Asian countries consists of multi-ethnical groups with possibly different inherent risk of developing ASVD, as well as the absence of risk estimators in population below 40 years of age much evidence is required to develop population-specific risk estimators and risk guided decision-making tools for primary prevention among South Asians using robust study designs. Furthermore, considering the sub-optimal healthcare systems and the absence of expensive investigating facilities like CAC in low and middle-income countries of South Asia, the development of risk estimators for these populations also requires large population-based, ethnicity and age-specific data to develop such a tool using both Astro-CHARM and PCE. Low and middle-income countries are now overburdened with interventional procedures like angioplasty, and expensive methods of secondary prevention. Accurate risk assessment is the only solution in such resource-poor settings where primary prevention can be implicated through lifestyle modifications and statins among high-risk individuals. However, overestimation and misclassification of an individual as high risk has its own implications too, not only at the individual level but also at healthcare system level, therefore, accuracy of the risk stratification modality is crucial.

### Strengths and limitations

The biggest strength of the study is the use and comparison of both ASCVD estimators in non-cardiac Pakistani general population selected from the largest city of the country with diverse population sub-groups from the entire country thus providing data facsimile to general population of Pakistan.

Major limitations of the study lie in the non-probability snowball sampling technique and study design where lack of prospective follow-up cannot ensure the accuracy of these estimates. Furthermore, the data was taken from an urban setting and implications of the findings cannot be ascertained on the rural population.

## Conclusion

Despite the fact that our findings showed substantial differences in 10-year risk of ASCVD between Astro-CHARM and PCE, both calculators can be used to develop a new population and specific risk estimators for this South Asian sub-population. Our study provides the first step towards developing a risk assessment guided decision-making protocol for primary prevention of ASCVD in this population.

## Data Availability

The data for the current study are available on request.

## Abbreviations

ASCVD: Atherosclerotic cardiovascular diseases
Astro-CHARM: Astronaut cardiovascular health and risk modification
CAC: Coronary artery calcium
CTA: Computed tomography angiography
CVD: Cardiovascular disease
hs-CRP: High sensitive C-reactive protein
NICVD: National institute of cardiovascular diseases
PCE: Pooled cohort equation
USA: United Sates of America
